# Supplementary material for: Lymphocyte Phenotypes and Protein-Bound Uremic Toxins as Determinants of Clinical Outcomes in Hemodialysis Patients
Source: Int J Mol Sci. 2025 Oct 24;26(21):10376. doi: 10.3390/ijms262110376 (PMC12607633; doi:10.3390/ijms262110376)
Supplement: Supplementary file 1 [file ijms-26-10376-s001.zip › Supplementary materials.pdf]

Analysis of lymphocytes

Analysis by flow cytometry was implemented within 12 hours after collection of the samples of the blood. Lymphocyte surface receptors, representative of their senescence (CD45RA, CCR7, CD28, CD57 for T lymphocytes and IgD and CD27 for B lymphocytes), were evaluated and followed by further analysis, which determined the lymphocytes' subsets by using a cell counter (Navios Flow Cytometer, Beckman Coulter, Indianapolis, IN, USA). The conjugated antibodies used for blood sample staining were anti-CD45 PC7 J33 (IM3548U, Beckman Coulter), anti-CD3 FITC UCHT1 (A07746, Beckman Coulter), anti-CD3 PE UCHT1 (A07747, Beckman Coulter), anti-CD4 Pacific blue MEM-241 (PB-359-T100, EXBIO, Praha SA, Czechia), anti-CD8 PC5 B9.11 (A7758, Beckman Coulter), anti-CD45RA APC MEM-56 (1A-223-T100, EXBIO), anti-CCR7 PE 4B12 (1P-735-C100, EXBIO), anti-CD28 CD28.2 PE-EF610 (61-0289-42, ThermoScientific LSG, Waltham, MA, USA), anti-CD31 APC MEM05 (T5-273-T100, EXBIO), anti-CD57 FITC TB01 (1F-158-T100, EXBIO), anti-CD279 (PD1) EI12.2H7 (11-176-C100, EXBIO), anti-CD19 PC5 J3-119 (Beckman Coulter), anti-IgD IA6-2 (Thermo Scientific LSG), and anti-CD27 PE-DyLight 594 (EXBIO).

CD4 and CD8 T cells were assorted in the following categories:

Early differentiated: recent thymic emigrants (RTEs), CD4+CD45RA+CD31+ and CD8+CD45RA+CD31+; naïve cells, CD4+CD45RA+CCR7+, CD4+CD28+CD57-, CD8+CD45RA+CCR7+, and CD8+CD28+CD57-.

Memory cells: central memory (CM) cells, CD4+CD45RA-CCR7+ and CD8+CD45RA-CCR7+; effector memory (EM) cells, CD4+CD45RA-CCR7- and CD8+CD45RA-CCR7-.

Advanced differentiated, senescent cells: effector memory re-expressing CD45RA cells (EMRA), CD4+CD45RA+CCR7-, CD8+CD45RA+CCR7-, CD4+CD45RA-CD57+, CD8+CD45RA-CD57+, CD4+CD28+CD57+, and CD8+CD28+CD57+.

Terminally differentiated, senescent cells: EMRACD28- (CD4+CD45RA+CCR7-CD28- and CD8+CD45RA+CCR7-CD28-), CD4+CD28-CD57+, and CD8+CD28-CD57+.

B cells were categorized as naïve (IgD+CD27-), IgM memory (IgD+CD27+), switched memory (IgD-CD27+), and double negative (IgD-CD27-).

Figures S1 and S2 describe the gating strategies of the T and B lymphocyte subpopulations.

Analysis of protein bound uremic toxins

The blood preparation for total protein bound uremic toxins concentration was as follows: 100 μL of plasma was diluted with 260 μL of ultra performance liquid chromatography grade water (Thermo Scientific, Geel, Belgium), for heat deproteinization, the samples were placed at 95 °C for 30 minutes cooled in an ice bath for 10 minutes, and centrifuged at 18,000×g for 10 minutes. The supernatant was centri-fuged through a 30 kDa cutoff centrifugal filter (Amicon Ultra 0.5, Merck KGaA, Darmstadt, Germany) for 20 minutes at 4500×g. For the free protein bound uremic toxins concentration, 260 μL of untreated plasma was centrifuged through a 30 kDa cutoff centrifugal filter at 4500×g for 20 minutes, and 100 μL of the ultrafiltrate was diluted with 260 μL of UPLC-grade water followed by the

same heat treatment as described. Ultimately, 180 µL of the ultrafiltrate was transferred into a vial, and internal standard (fluorescein; 50 ppm) was added. Ultra performance liquid chromatography (Agilent 1290 Infinity device; Agilent, Santa Clara, CA, USA) was used to separate the protein bound uremic toxins. Hippuric acid and 3-carboxy-4-methyl-5-propyl-2-furanpropionate were detected with an Agilent G4212A diode array detector at 245 nm and 254 nm, respectively. Indoxyl sulfate (λex: 280 nm, λem: 376 nm), p-cresyl sulfate and p-cresyl glucuronide (λex: 264 nm, λem: 290 nm), indole-3-acetic acid (λex: 280 nm, λem: 350 nm), and fluorescein (λex: 443 nm, λem: 512 nm) were detected by an Agilent G1316C fluorescence detector.
