# Supplementary material for: Lymphocyte Phenotypes and Protein-Bound Uremic Toxins as Determinants of Clinical Outcomes in Hemodialysis Patients
Source: Int J Mol Sci. 2025 Oct 24;26(21):10376. doi: 10.3390/ijms262110376 (PMC12607633; doi:10.3390/ijms262110376)
Supplement: Supplementary file 1 [file ijms-26-10376-s001.zip › Supplemental figure legends.pdf]

Supplemental figure legends

Supplemental Figure S1. Gating strategy for CD4 and CD8 cells and their subsets, based on the presence of CD45RA, CCR7, CD31, CD57, and CD28

Supplemental Figure S2. Gating strategy for CD19+ cells and their subsets based on the presence of IgD and CD27
